# Microsporum gypseum Infection Among Two Related Families With a Zoonotic Aspect: A Prospective Case Series

**DOI:** 10.7759/cureus.51402

**Published:** 2023-12-31

**Authors:** Faisal Hassan Tobeigei, Martin R Joseph, Ahmed Al-Hakami, Mohamed E Hamid

**Affiliations:** 1 Dermatology Division, Department of Internal Medicine, College of Medicine, King Khalid University, Abha, SAU; 2 Department of Clinical Microbiology and Parasitology, King Khalid University, Abha, SAU; 3 Department of Clinical Microbiology and Parasitology, College of Medicine, King Khalid University, Abha, SAU

**Keywords:** aseer region, itraconazole, dermatophytes, zoonosis, tinea, dermatophytosis

## Abstract

Background and purpose

The *Microsporum gypseum *complex is a globally distributed group of geophilic dermatophytes that primarily affect animals but can also rarely cause dermatomycoses in humans. With some regional and occupational variations, tinea corporis is the most prevalent presentation of the infection. The aim of this study was to report on the diagnosis and treatment of dermatophytosis cases among related families, and their pets, from southern Saudi Arabia. Up-to-date information on dermatophytes and dermatophytosis is needed.

Methods

This is a prospective case series undertaken at the Dermatology Outpatient Clinic of King Khalid University, Saudi Arabia. Six patients with suspected dermatophytosis were received at our hospital in 2022 and have been followed for recovery with or without scars. Characteristics of fungal pathogens were examined phenotypically on the basis of microscopic and growth characteristics, and laboratory data were used to initiate treatment with oral fluconazole, topical terbinafine cream, or oral itraconazole.

Results

Clinical features and culture results confirmed tinea capitis and tinea corporis caused by *M. gypseum*, which was also present in a pet cat. Tinea capitis cases (n = 4) did not respond to fluconazole and terbinafine treatment, but treatment with itraconazole resulted in a full recovery. Tinea corporis cases (n = 2) were treated with terbinafine, which resulted in a full recovery within four weeks, with no signs of scarring.

Conclusions

*M. gypseum* presents with serious persistent lesions and is extremely contagious. Treatment is durable but challenging, and breaking the transmission chain is more difficult.

## Introduction

Tinea or dermatophytosis refers to a group of diseases of the scalp, hair, body, and surrounding skin infections caused by dermatophyte species. Dermatophytes are a set of filamentous fungal species that affect the skin [1]. They include anthropophilic, zoophilic, and geophilic types. The most commonly isolated species in tinea capitis include *Microsporum canis* (80%), *Trichophyton tonsurans* (15%), and other species (5%), such as *Trichophyton violaceum*, *Trichophyton rubrum*, *Trichophyton mentagrophytes*, and *Microsporum​​​​​​​ gypseum*. At present, *T. rubrum* is the leading pathogen in skin and nail infections while *M. canis*, *T. tonsurans*, and *T. violaceum* prevail at the scalp [1-3].

Dermatophytosis mainly occurs in warm and humid climates in tropical regions and predominantly affects children; it is rare in infants in the first year of life [4-6]. The causal agent and disease occurrence differ according to environmental and topographical areas. Infections due to *M. gypseum* usually present as an inflammatory mycosis that normally affects glabrous skin and scalp, notably in children. Not often, it can cause onychomycosis [7]. Most dermatophytic infections are diagnosed clinically, but when steroid ointments and creams are applied topically, they are frequently mistaken for other skin infections, resulting in additional misdiagnosis and poor treatment. As a result, accurate, effective, and quick laboratory diagnosis of dermatophytes becomes necessary. Usually, the macroscopic observation of colony morphology (pigmentation, growth rate, texture, etc.) produced on selective media is used to identify dermatophytes in the laboratory. This is followed by a microscopic study of conidia [1].

Treatment regimens are required since *M. canis* infections are very infectious and can potentially spread to other receptive hosts. Many topical and oral antifungal medications are available, and medications like fluconazole, terbinafine, itraconazole, and griseofulvin are used to treat serious infections in both humans and animals. However, 25-40% of treated patients have *M. canis* infections that are characterized by recurrence and treatment failure. This could be because of resistance phenomena, variable medication absorption, poor patient compliance, and drug penetration into tissue [2,3].

In Saudi Arabia, children under the age of 10 years were the most vulnerable, followed by adults between the ages of 31 and 40 years. The most prevalent causal agent was unicellular yeast, with *T. tonsurans* and *M. canis* coming in second and third, respectively, as the etiological agents [4]. Children and adolescents were commonly affected by dermatophytosis, with hair and scraping being the most prevalent specimens. Over time, the species of *Trichophyton* and *Microsporum* decreased significantly [5]. There have been reports of a comparatively high frequency of fungal species in the carpets of mosques in the Riyadh region. The study recommended that new policies and tactics be put in place to raise awareness of hygiene [6].

Dermatophytes and dermatophytosis may be showing transformations in biological comportment, and there is a tendency for the diagnosis to be neglected [8]. Accordingly, clinical and laboratory studies are needed to address out-of-date, missing, and deficient epidemiological, diagnostic, and treatment knowledge. Herein, we describe a case series of dermatophytosis caused by *M. gypseum* from patients in southern Saudi Arabia's Aseer region.

## Materials and methods

Setting and patients

We performed a prospective study at the Dermatology Outpatient Clinic of King Khalid University Hospital Polyclinics and the Department of Microbiology, College of Medicine, King Khalid University, Abha, Saudi Arabia.

Six mycologically proven tinea cases treated at our hospital in 2022 were identified. The patients presented with skin lesions indicative of tinea infection. The patients were from the Aseer region, Southern Saudi Arabia. All patients and their pet cats were examined clinically and underwent sampling and treatment accordingly.

Specimen collection

Sample collection and processing were done following standard procedure [7]. Sampling was done with a sterile scalpel blade or curette. The lesion area to be sampled was disinfected using 70% ethyl alcohol to eliminate dirt and contaminating bacteria. Skin scales, crusts, or biopsy samples were collected from the erythematous, peripheral, and actively growing margins of the lesions by scraping through the inflamed margin of the lesion into the seemingly healthy tissue using the dull side of a sterile surgical blade onto clean glass slides. Hair specimens were collected by using epilating forceps. Hair samples from skin lesions of two cats with skin lesions were collected to determine the diagnosis. Specimens were stored in sterile dry Petri dishes and then transported to the laboratory for mycological examination. The samples were divided into two portions: one for microscopic investigation and one for culturing.

Laboratory diagnosis

The diagnosis of tinea was established by direct microscopy using a 10% potassium hydroxide mount and culturing on Sabouraud dextrose agar (SDA) plates. Fungal cultures were handled at the Microbiology Laboratory at the College of Medicine, King Khalid University. Skin scrapings, hairs, and biopsy samples were inoculated onto SDA and Mycosel agar plates (Oxoid, London, UK). The plates were inoculated and then incubated at 25°C for up to four weeks, with daily checking for growth or contamination. Suspect colonies were confirmed by microscopic examination.

Identification of fungi

Identification of fungi was performed based on the appearance of colony morphology on SDA and microscopic structures as per the guiding principles [9]. Suspected molds were sub-cultured on new SDA plates to enhance the growth and development of characteristic hyphae and conidial elements. Grown mold cultures were investigated macroscopically for the color of the surface and reverse, features, and consistency, as well as microscopically for fungal elements such as spores, hyphae, and conidia. After four weeks of incubation, if there was no growth, the culture was considered negative. All isolated fungi were identified at no less than the genus level.

Treatment

Subsequent to mycological confirmation, patients were initially treated with oral fluconazole (Diflucan, Pfizer) and the dose was 6 mg/kg body weight, and topical terbinafine cream (1%). Subsequently, the treatment was shifted to oral itraconazole (Tracon, SPIMACO, Al-Qassim Pharmaceutical Plant, Saudi Arabia) and the dose was 5 mg/kg body weight per day at a dose of 5 mg/kg/day for three months.

Clinical treatment was confirmed by mycological examination. Epidemiological and clinical data were collected for all cases.

## Results

Demographic, clinical, and laboratory findings of the six patients with mycologically proven tinea are shown in Table 1. The patients appeared with skin lesions ranging from multiple hairless erythematous plaques, sometimes with pain, to scalp abscesses.

**Table 1:** Patient demographic, clinical, and laboratory data and treatment outcomes.

Case number	Age/sex	Relationships and contact	Comorbidity	Clinical presentation	Diagnosis	Culture isolates	First-line treatment	Second-line treatment
1	4 years/female	Two sisters and a brother contact with an infected domestic cat	None	Multiple hairless erythematous plaques	Tinea capitis	Microsporum gypseum	Oral fluconazole and topical terbinafine, without improvement	Oral itraconazole (5 mg/kg per day)/full recovery
2	25 years/female			Scaly erythematous annular plaque on the right flank	Tinea corporis	Microsporum gypseum	Topical terbinafine/6 weeks; full recovery without scarring	N/A
3	21 years/male			Scaly erythematous plaque on the left anterior part of the neck	Tinea corporis	Microsporum gypseum	Topical terbinafine/6 weeks; full recovery without scarring	N/A
4	10 years/male	Brothers contact with relatives (cases 1-3). Infrequent contact with the infected cat in their relative‘s house		Multiple painful scalp abscesses	Tinea capitis	Microsporum gypseum	Oral fluconazole/4 weeks, without improvement	Oral itraconazole (5 mg/kg per day)/3 months; recovery from tinea (with mild atrophic scars)
5	8 years/male			Multiple scaly plaques on the scalp without abscesses	Tinea capitis	Microsporum gypseum	Oral fluconazole/4 weeks, without improvement	Oral itraconazole (5 mg/kg per day)/3 months; recovery from tinea (with mild atrophic scars)
6	6 years/male		None	Multiple scaly plaques on the scalp without abscesses	Tinea capitis	Microsporum gypseum	Oral fluconazole/4 weeks, without improvement	Oral itraconazole (5 mg/kg per day)/3 months; recovery from tinea (with mild atrophic scars)

Samples from the cats showed no fungal elements when 10% KOH preparations were examined microscopically. The cultures on SDA were positive for *M. gypseum* from the cat’s samples. The detailed results of the six human cases were as follows.

Case number 1: tinea capitis

**Figure 1:**
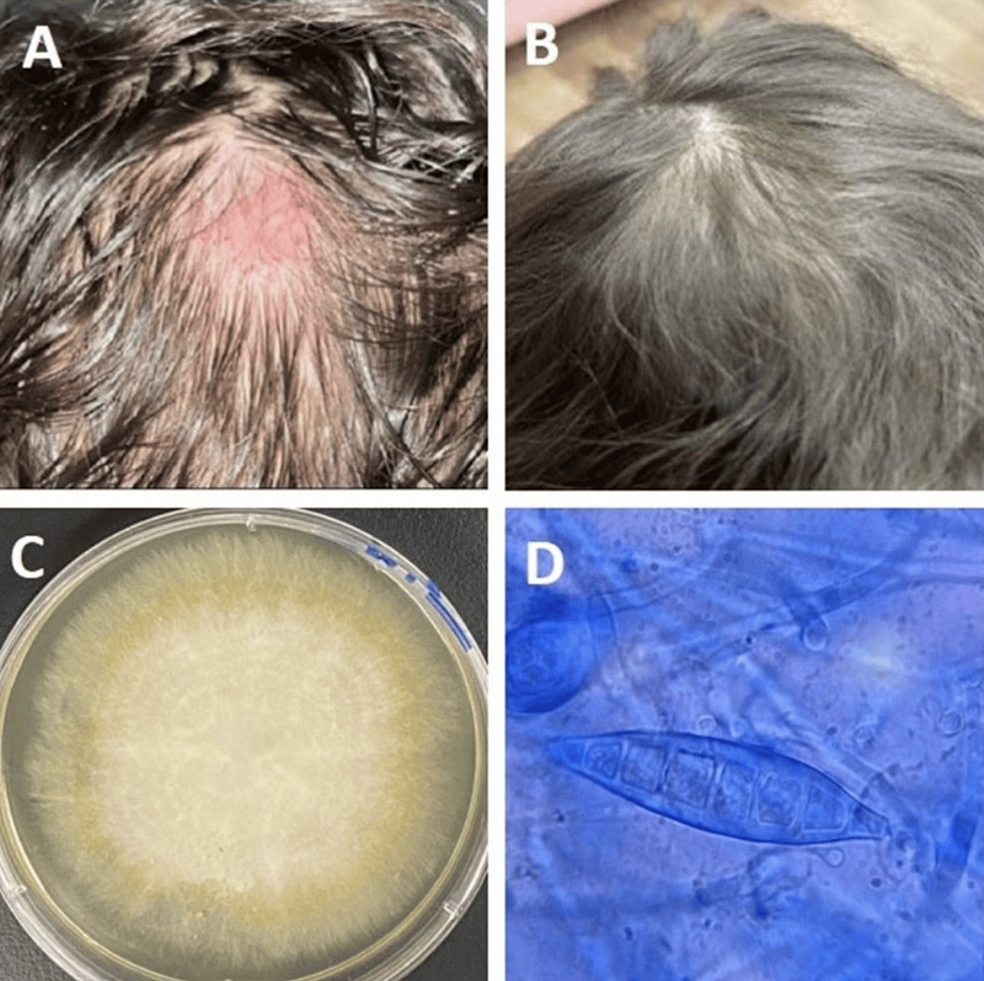
(A) A four-year-old girl with an erythematous (itchy) annular hairless plaque on the scalp at the vertex. (B) Full recovery after itraconazole treatment. (C) Growth of the mold on Sabouraud dextrose agar. (D) A smear from grown fungus reveals septate fusiform thin-walled macroconidia with three to six cells.

A four-year-old girl attended our Dermatology Clinic with an itchy erythematous annular hairless plaque on the scalp at the vertex (Figures 1A, 1B). Contact with a cat of a friend of the family two weeks prior was affirmed. Before she visited our department, she received many topical treatments, including steroids as a case of alopecia areata; however, she became worse.

Fungal cultures of both the hair and scalp showed the growth of *M. gypseum*. The colonies on SDA were flat, scattering, and white to cream-colored, with a dense cottony surface and radial grooves (Figure 1C). Colonies generally were light golden-yellow to brownish-yellow at the reverse side of the plate; however, non-pigmented strains were also observed. Fungal elements with septate hyphae with conidia (macroconidia and microconidia) were observed microscopically. The macroconidia were plentiful, fusiform, and regular in shape, with rounded ends. The walls of macroconidia were thin and rough and contained three to six cells (Figure 1D).

Oral fluconazole (6 mg/ kg) and topical terbinafine cream (1%) were initiated; however, there was no improvement after four weeks. Therefore, oral itraconazole (calculated according to her weight as 5 mg/kg per day) was initiated, which led to a full recovery and regrowth of hair, without scarring, after three months of treatment.

Case number 2: tinea corporis

**Figure 2:**
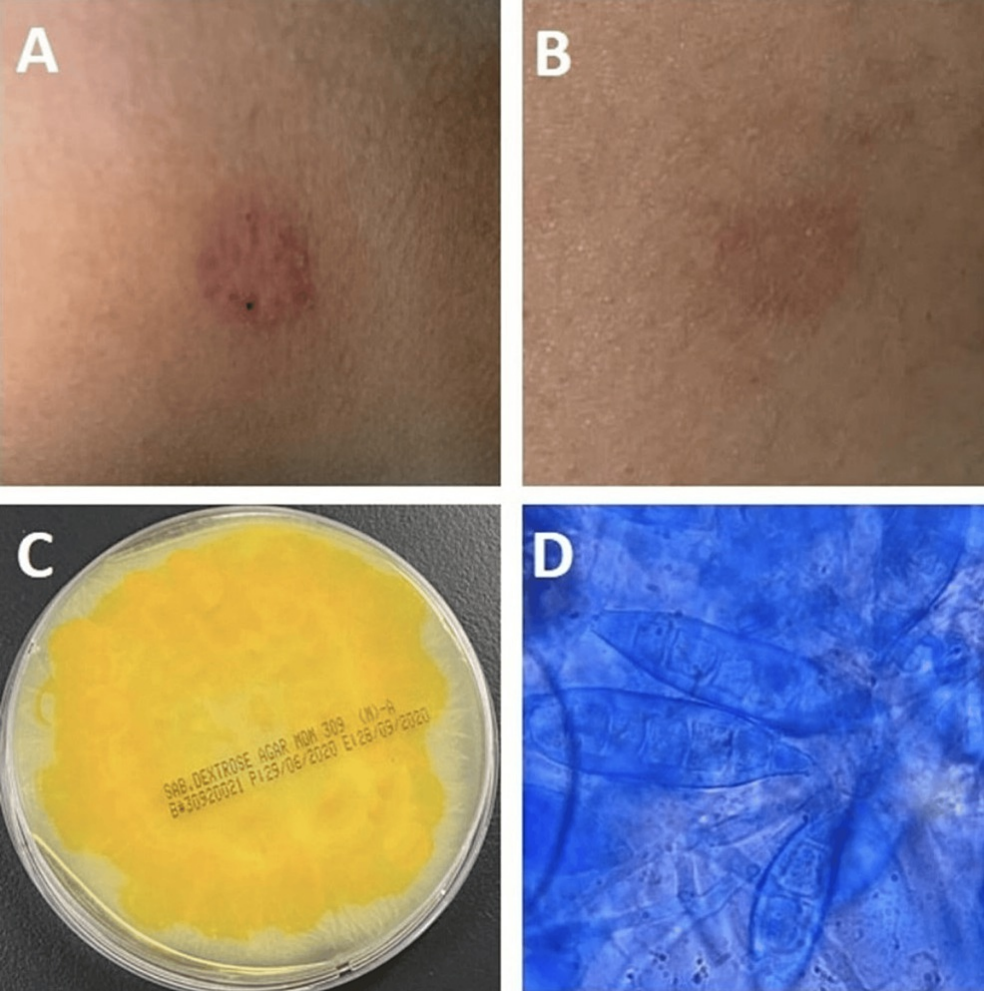
(A, B) A 25-year-old woman with scaly erythematous annular plaque on her right flank. (C) The reverse of cultured plates had a dark yellow color. (D) A microscopic image of a smear from the grown culture reveals a cluster of septate fusiform thin-walled macroconidia with three to six cells, characteristic of Microsporum gypseum.

A 25-year-old woman (sister of case number 1) presented with a scaly erythematous annular plaque on her right flank (Figures 2A, 2B). Fungal cultures and microscopy confirmed *M. gypseum*. She was treated with topical terbinafine cream (1%), and within six weeks, she was fully healed, without scarring.

The colonies on SDA were flat, scattered, and white to cream-colored, and showed radial grooves. Colonies were generally bright golden-yellow to brownish-yellow on the reverse side of the plate (Figure 2C). Microscopically, septate hyphae with conidia (macroconidia and microconidia) were observed under the microscope. The macroconidia were abundant, fusiform, and symmetrical in shape, with rounded ends, and contained three to six cells (Figures 2C, 2D).

Case number 3: tinea corporis

**Figure 3:**
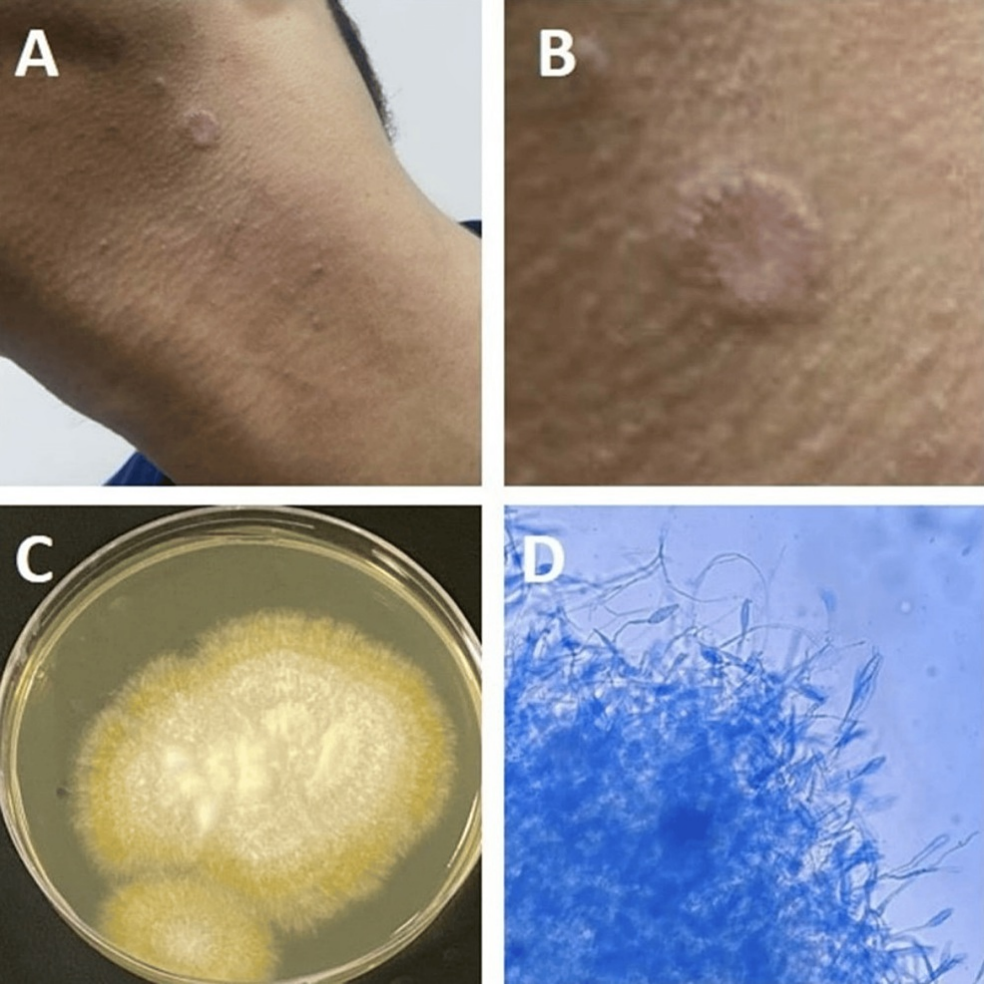
(A, B) A 21-year-old man presented with a scaly erythematous annular plaque on the neck. (C) The reverse of cultured plates had a dark yellow color. (D) A microscopic image of a smear made from the grown culture reveals a cluster of septate fusiform thin-walled macroconidia with three to six cells, characteristic of Microsporum gypseum.

A 21-year-old man (brother of case number 1) presented with a scaly erythematous plaque on the left anterior part of the neck (Figure 3). Fungal culture and microscopy confirmed *M. gypseum*. He was treated with topical terbinafine cream (1%), and within six weeks, he was fully healed, without scarring.

Case number 4: tinea capitis

**Figure 4:**
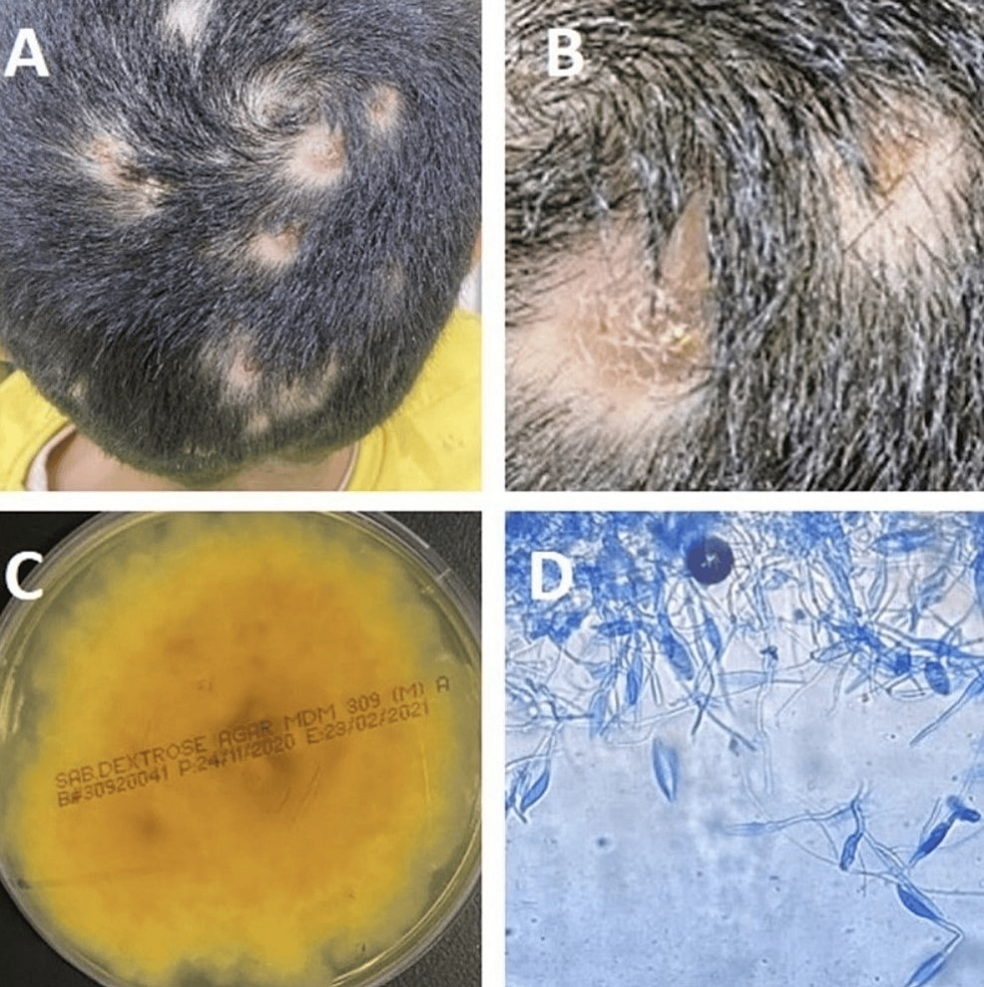
(A, B) A 10-year-old boy with multiple painful scalp abscesses. (C) The reverse of a cultured plate had a dark yellow color. (D) A microscopic image of a smear from the grown culture reveals a cluster of septate fusiform thin-walled macroconidia with three to six cells, characteristic of Microsporum gypseum.

A 10-year-old boy presented with severe multiple scalp abscesses, which had lasted for more than five weeks and increased under topical treatment (Figures 4A, 4B). The patient and his parents affirmed contact with a cat in their relative’s home. A swab from the abscesses confirmed the growth of *M. gypseum*. The colonies on SDA were flat, scattered, and white to cream-colored, and showed radial grooves. The colonies were generally bright golden-yellow to brownish-yellow on the reverse side of the plate (Figure 4C). Under microscopy, the smear from fungal colonies showed septate hyphae with abundant fusiform and symmetrical macroconidia and microconidia, which contained three to six cells (Figure 4D).

Systemic treatment with oral fluconazole (6 mg/ kg) for approximately four weeks failed to result in any improvement. Thus, the medication was changed to oral itraconazole (5 mg/kg per day). Three months of continuous intake of itraconazole led to a full recovery with mild atrophic scars.

Case number 5: tinea capitis

**Figure 5:**
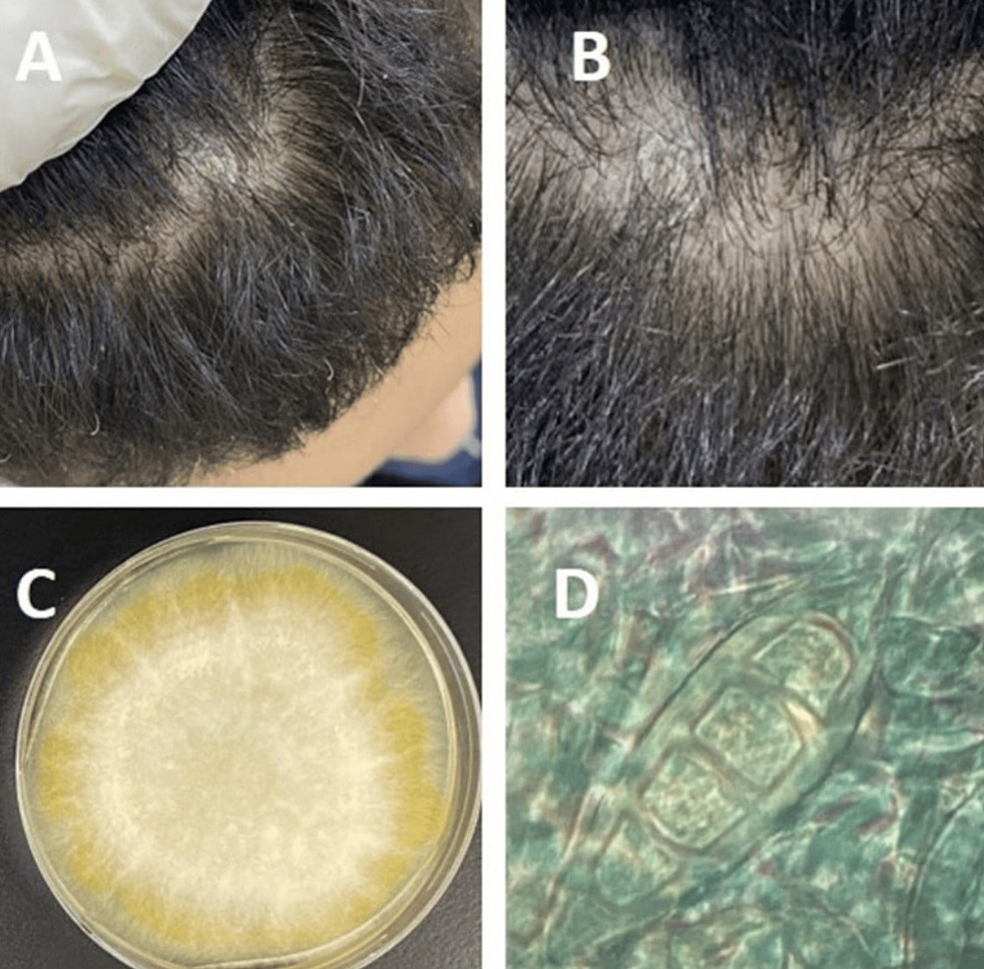
(A, B) An eight-year-old boy with multiple scaly plaques on the scalp without abscesses. (C) The reverse of the cultured plate had a dark yellow color. (D) A microscopic image of a smear from the grown culture reveals a cluster of septate fusiform thin-walled macroconidia with three to six cells, characteristic of Microsporum gypseum.

An eight-year-old boy reported to our clinic with severe multiple scaly plaques on the scalp without abscesses, which had lasted for more than five weeks and increased under topical treatment (Figures 5A, 5B). The patient and his parents confirmed contact with a cat in their relative’s house. A swab from the abscesses of this patient confirmed the growth of *M. gypsum* on culture. The colonies were uniform, distributed, and gray to cream-colored, with a thick cottony surface and radiating grooves. The colonies were generally bright golden-yellow to brownish-yellow on the reverse side of the plate (Figure 5C). Microscopically, fungal elements with many septate hyphae and fusiform macroconidia and microconidia were observed microscopically. The macroconidia had symmetrical round ends, with thin and rough walls containing three to six cells (Figure 5D).

Systemic treatment with oral fluconazole (6 mg/kg per day) for approximately four weeks failed to result in any improvement. Thus, the medication was changed to oral itraconazole (5 mg/kg per day). Three months of continuous intake of itraconazole led to a full recovery with mild atrophic scars.

Case number 6: tinea capitis

**Figure 6:**
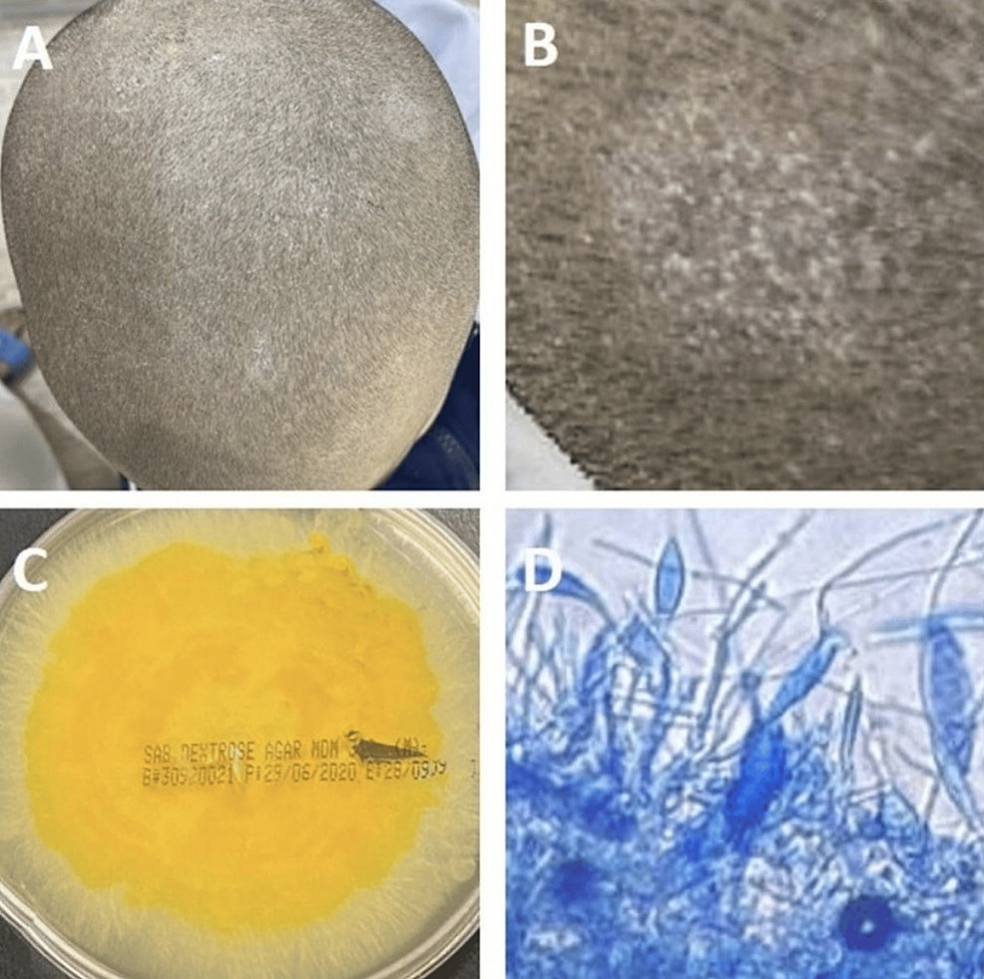
(A, B) A six-year-old boy with multiple scaly plaques on the scalp without abscesses. (C) The reverse of the cultured plates had a dark yellow color. (D) A microscopic image of a smear from the grown culture reveals a cluster of septate fusiform thin-walled macroconidia with three to six cells, characteristic of Microsporum gypseum.

A six-year-old boy presented with multiple scaly plaques on the scalp without abscesses, which had lasted for more than five weeks and increased under topical treatment (Figures 6A, 6B).

The patient and his parents affirmed occasional contact with a cat in their relative’s home. A swab from the abscesses confirmed the presence of *M. gypsum* using both culture and microscopy. The colonies on SDA were flat, scattered, and white to cream-colored, and exhibited radial grooves. The colonies were generally bright golden-yellow to brownish-yellow on the reverse side of the plate (Figure 6C). Microscopically, septate hyphae with conidia (macroconidia and microconidia) were observed under the microscope. The macroconidia were plentiful, fusiform, and symmetrical in shape with round ends and contained three to six cells (Figure 6D).

Systemic treatment with daily oral fluconazole (6 mg/kg per day) for approximately four weeks failed to result in any improvement. Thus, the medication was changed to oral itraconazole (5 mg/kg per day). Three months of continuous intake of itraconazole led to a full recovery with mild atrophic scars.

## Discussion

Dermatophytosis is a disease of international importance and a public health problem. The prevalence of superficial mycotic infection is 20-25% worldwide, with dermatophytes as the most common agent [8]. Further, dermatophytosis is a major public health challenge in tropical and subtropical countries such as Saudi Arabia [9]. Tinea pedis, onychomycosis, tinea cruris, and tinea capitis are the most frequent dermatophyte infections and affect all socioeconomic groups [10]. Children in schools mostly become infected owing to various predisposing factors, especially overcrowding [11]. These infections remain largely neglected and out of control in many rural areas with low socioeconomic status or pastoral activities [12].

Cutaneous mycoses, especially those caused by dermatophyte fungi, are among the most common fungal infections. More than 10% of the population will be infected by a dermatophyte sometime in their lifetime [13]. Therefore, reliable laboratory approaches that are accessible are needed to enable a prompt and accurate diagnosis, enabling correct medicine and prevention procedures.

In the current study, six clinically suspected cases of tinea infections were observed over a period of 12 months. The tinea types comprised tinea capitis (n = 4) and tinea corporis (n = 2). Tinea capitis cases were treated with oral itraconazole, whereas tinea corporis cases were treated with topical terbinafine. Initial treatment of tinea capitis with fluconazole consistently failed after four weeks of treatment. Variation in treatment response has been reported in the literature, necessitating the implementation of early clinical and laboratory assessment along with epidemiological information. Consistent with the treatment outcomes in the present study, itraconazole oral solution (pulse therapy) has been noted as a promising treatment for tinea capitis in infants [14,15]. However, other reports noticed that the management of *Microsporum* spp. tinea capitis using pulsed oral terbinafine is very successful [16].

The disease and its etiology appear to be transforming in terms of biological nature and affinity to treatment; however, a diagnosis is possible at an early stage if necessary steps are undertaken promptly [17]. Physicians should be attentive to the clinical range of mycotic infections caused by *Microsporum* spp. to avoid errors in identifying the fungi and deliver the correct therapy to patients [18]. Although tinea capitis in adults is considered rare, some reports indicate a significant increase in cases [19]. Further, it has been reported that tinea capitis is often misdiagnosed, as the lesions can simulate dandruff and seborrhea [20].

The management of tinea includes the use of topical antifungals and oral medications; the latter is especially needed in widespread cases. During the last decade or two, there has been a steady rise in the incidence of chronic dermatophyte infections of the skin and the occurrence of problems with treatment. It is noteworthy that there are no standardized national or international guidelines on the management of tinea. However, treatment with systemic antifungals is often empirical [8].

Another factor contributing to challenges in treatment is the fact that the clinical presentation of tinea varies for numerous reasons; for example, the causal organism, host inflammatory response, and immune status, age, and sex of the host [21]. This circumstance is specifically frequent in the case of zoophilic infections [22]. Tinea capitis may well initiate as a non-inflammatory black spot on the scalp before hair shafts over the area begin to weaken and hairs detach. Ultimately, a distinctive zone of hair loss is formed. Other types of infection models comprise non-inflammatory seborrheic dermatitis, and in certain carriers, tinea capitis may be asymptomatic [21].

The small sample size in this report is one of the limitations of the study. Also, the follow-up of the pet cats was found difficult to reach and no treatment has been initiated.

## Conclusions

*M. gypseum* presents with serious lesions and challenges in its treatment, and animal transmission is evident. Generally, dermatophytosis is widespread in southern Saudi Arabia, given the hot and humid climate, which promotes the growth of fungi. The present report emphasizes the significance of the mycological analysis in the diagnosis of dermatophytosis for efficient treatment, especially in children. Tinea capitis cases did not respond to fluconazole and terbinafine treatment after four weeks of treatment, whereas tinea corporis cases were successfully treated with topical terbinafine.
